# Baseline D-Dimer Levels as a Risk Assessment Biomarker for Recurrent Stroke in Patients with Combined Atrial Fibrillation and Atherosclerosis

**DOI:** 10.3390/jcm8091457

**Published:** 2019-09-13

**Authors:** Kang-Ho Choi, Woo-Keun Seo, Man-Seok Park, Joon-Tae Kim, Jong-Won Chung, Oh Young Bang, Geong-Moon Kim, Tae-Jin Song, Bum Joon Kim, Sung Hyuk Heo, Jin-Man Jung, Kyungmi Oh, Chi Kyung Kim, Sungwook Yu, Kwang Yeol Park, Jeong-Min Kim, Jong-Ho Park, Jay Chol Choi, Yang-Ha Hwang, Yong-Jae Kim

**Affiliations:** 1 Department of Neurology, Chonnam National University School of Medicine and Hospital, Gwangju 61469, Korea; mspark@chonnam.ac.kr (M.-S.P.); alldelight2@chonnam.ac.kr (J.-T.K.); 2 Department of Neurology, Samsung Medical Center, Sungkyunkwan University School of Medicine, Seoul 06351, Korea; neurocjw@gmail.com (J.-W.C.); ohyoung.bang@samsung.com (O.Y.B.); gyeongmoon.kim@samsung.com (G.-M.K.); 3 Department of Neurology, Ewha Womans University, Seoul 03760, Korea; knstar@hanmail.net; 4 Department of Neurology, Kyung Hee University College of Medicine, Seoul 02447, Korea; medicj80@daum.net (B.J.K.); shheo73@hanmail.net (S.H.H.); 5 Department of Neurology, Korea University Ansan Hospital, Korea University College of Medicine, Kyungki-Do 15355, Korea; sodium75@hanmail.net; 6 Department of Neurology, Korea University Guro Hospital, Korea University College of Medicine, Seoul 08308, Korea; okyungmi@korea.ac.kr (K.O.); ckkim7@gmail.com; (C.K.K.); 7 Department of Neurology, Korea University Hospital, Korea University College of Medicine, Seoul 02841, Korea; song4yu@korea.ac.kr; 8 Department of Neurology, Chung-Ang University College of Medicine, Seoul 06974, Korea; kwangyeol.park@gmail.com (K.Y.P.);; 9 Department of Neurology, Myongji Hospital, Hanyang University College of Medicine, Goyang 10475, Korea; neurotector.jhp@gmail.com; 10 Department of Neurology, Jeju National University, Jeju 63241, Korea; jaychoi@jejunu.ac.kr; 11 Department of Neurology, Cerebrovascular Center Kyungpook National University School of Medicine and Hospital, Daegu 41944, Korea; yangha73@gmail.com; 12 Department of Neurology, Eunpyeong St. Mary’s Hospital, The Catholic University of Korea, Seoul 03312, Korea; brain930@ewha.ac.kr

**Keywords:** atrial fibrillation, d-dimer, outcome, ischemic stroke, antithrombotics

## Abstract

Background: We investigated the effect of D-dimer levels and efficacy of different antithrombotic therapies according to the baseline D-dimer levels on recurrent stroke in patients with atrial fibrillation (AF)-related stroke and atherosclerosis. Methods: We enrolled 1441 patients with AF-related stroke and atherosclerosis in this nationwide multicenter study. The primary outcome measure was the occurrence of recurrent ischemic stroke over a 3-year period. Results: High D-dimer levels (≥2 μg/mL) were significantly associated with higher risk of recurrent ischemic stroke (adjusted hazard ratio (HR), 1.80; 95% confidence interval (CI), 1.13–2.84; *p* = 0.012). The risk of recurrent stroke was similar between the anticoagulant and the antiplatelet groups in all subjects (adjusted HR, 0.78; 95% CI, 0.46–1.32; *p* = 0.369). However, in patients with high D-dimer levels (≥2 μg/mL), risk of recurrent stroke was significantly lower in the anticoagulant group than in the antiplatelet group (adjusted HR, 0.40; 95% CI, 0.18–0.87; *p* = 0.022). Conclusion: Our findings suggested that baseline D-dimer levels could be used as a risk assessment biomarker of recurrent stroke in patients with AF-related stroke and atherosclerosis. High D-dimer levels would facilitate the identification of patients who are more likely to benefit from anticoagulants to ensure secondary prevention of stroke.

## 1. Introduction

Stroke is a heterogeneous disease with multiple risk factors and causes [1]. Secondary prevention of recurrent ischemic stroke targets the mechanisms responsible for the stroke index [2]. Several studies have recommended the use of antiplatelet agents instead of oral anticoagulants (OACs) to address atherosclerosis-associated non-cardioembolic stroke, but OACs for cardioembolic stroke during secondary prevention [3]. However, several patients either demonstrated both cardioembolic and non-cardioembolic etiologies or are at a high risk of concomitant cardio- and peripheral-atherosclerotic diseases and recurrent cardioembolic stroke [4]. Although guidelines recommend OACs for patients with atrial fibrillation (AF) and stroke, including those with atherosclerotic diseases, there is insufficient evidence to successfully identify which antithrombotic therapies are more effective for secondary prevention of stroke in patients with AF and atherosclerosis [3]. Furthermore, a tailored antithrombotic strategy based on a risk assessment biomarker for recurrent stroke in these patients has not yet been established [1].

Previous reports suggest that plasma D-dimer levels—a thrombin generation and fibrin turnover biomarker—may be a clinically useful risk assessment biomarker for stroke in patients with AF [5]. Higher baseline D-dimer levels were associated with increased frequencies of stroke in AF patients [6,7]. The efficacy of anticoagulants for stroke prevention in AF patients could be related to the suppression of elevated D-dimer levels. Similarly, the superior ability of non-vitamin K antagonist OACs (NOACs) to further reduce elevated D-dimer levels compared to warfarin might be attributed to the relatively higher efficacy of NOACs [7,8]. However, there are no reports that have examined the effect of D-dimer levels or proper antithrombotic strategies according to baseline D-dimer levels for secondary prevention of stroke in patients with AF and atherosclerosis.

Therefore, we investigated the impact of D-dimer levels and the efficacy of different antithrombotic therapies according to the baseline D-dimer levels on the prevalence of recurrent stroke in a nationwide population of patients with AF-related stroke and atherosclerotic diseases.

## 2. Materials and Methods

### 2.1. Subjects

To conduct this retrospective study, we used data from a prospective, nationwide, multicenter registry the Korean Atrial Fibrillation Evaluation Registry in Ischemic Stroke Patients (K-ATTENTION)—a database of consecutive acute ischemic stroke (AIS) patients with AF who were admitted to 11 medical centers in South Korea between January 2013 and December 2015. Among the 3213 patients registered in the database, we included those who met the following conditions: (1) presence of atherosclerotic vascular diseases (ischemic stroke presumed arterial origin or atherosclerosis in the carotid, intracranial, coronary, or peripheral arteries), and (2) patients taking antithrombotic agents at discharge. Patients were excluded if they (1) had incomplete data for D-dimer levels, (2) had incomplete data for clinical outcomes over a 3-year period, and (3) were diagnosed with AF after discharge.

The impact of D-dimer levels on recurrent stroke was investigated by categorizing the study subjects into groups based on prespecified baseline D-dimer levels (<0.5 μg/mL vs. ≥0.5 μg/mL; <1 μg/mL vs. ≥1 μg/mL; <2 μg/mL vs. ≥2 μg/mL; <3 μg/mL vs. ≥3 μg/mL). Principally, the D-dimer levels were noted during the first assessment immediately after admission, according to the local practice of each center, i.e., either immediately in the emergency room at admission or in the days following admission.

We further categorized the subjects into groups based on the antithrombotic agents prescribed upon discharge (antiplatelets alone vs. OACs with or without antiplatelets; warfarin vs. NOACs; dual antithrombotics vs. OACs alone). We analyzed the efficacy of antithrombotic regimens for secondary prevention of recurrent stroke and their safety according to baseline D-dimer levels on the stroke index in the population that survived and underwent antithrombotic treatment both during hospitalization and at discharge. Additionally, we concomitantly compared the results of the enrolled patients with atherosclerosis with the results of those without atherosclerosis and those who did not meet the inclusion criteria, to assess the likelihood of selection bias. The antithrombotics were administered at the discretion of the treating physician. We included all varieties of NOACs (dabigatran, apixaban, rivaroxaban, and edoxaban).

### 2.2. Ethics Statement

This study was approved by the institutional review boards of all participating centers. The necessity to obtain patient consent was waived since our procedures and protocols adequately protected their anonymity. All clinical and laboratory investigations described in this study were conducted according to the principles expressed in the Declaration of Helsinki. All supporting data within the article is available from the corresponding author on reasonable request.

### 2.3. Protocol and Outcome Measurements

The primary outcome measure was the occurrence of recurrent ischemic stroke according to baseline D-dimer levels and the antithrombotic therapies over a 3-year period. Recurrent ischemic stroke was defined as the sudden development of a new focal neurologic deficit or the worsening of a pre-existing one after the index stroke event, evidenced by the presence of new ischemic lesions on brain imaging (magnetic resonance imaging or computed tomography). Key secondary outcomes included the occurrence of any stroke, intracranial hemorrhage (ICH), acute coronary syndrome (ACS), and major extracranial bleeding. ACS was defined as myocardial infarction (MI), unstable angina, or ischemia-associated sudden cardiac death.

Clinical information for outcomes after discharge was prospectively obtained from all patients, either during their routine clinic visits or by corresponding with them or their caregivers over a 3-year period. Adherence to prescribed medication was assessed via interviews three months after the index event using the six-item modified Morisky Medication Adherence Scale at the largest center [9]. High adherence was defined as motivation and knowledge domain scores of ≥2. As recommended by current guidelines, left ventricular (LV) diastolic dysfunction was defined with an algorithm for grading LV diastolic function in patients with AF [10]. Congestive heart failure was defined as a clinical syndrome that was diagnosed by a cardiologist, having a New York Heart Association class II or higher [11]. Baseline clinical and laboratory data about the history of risk factors that were etiologically associated with atherosclerosis and AF, including hypertension, dyslipidemia, diabetes mellitus, obesity, and current smoking habits was collected from all patients [12].

### 2.4. Statistical Analysis

Differences between the groups based on the use of antithrombotic agents were analyzed using Student’s *t-*tests or the Kruskal–Wallis test for continuous variables. The chi-square (χ^2^) test or Fisher’s exact test was used to analyze categorical variables. A *p* value of <0.05 was considered statistically significant. Unadjusted associations for antithrombotic therapy and risks for clinical outcomes were obtained using Kaplan–Meier curves throughout the study period to account for censoring.

Cox proportional hazard regression models were subsequently constructed to calculate adjusted hazard ratios (HRs) and 95% confidence intervals (CIs), for clinical outcomes concerning antithrombotic therapy, according to the baseline D-dimer levels. Based on the clinical significance and the results of previous studies, several adjustments were made to incorporate information from the following variables: Age, sex, hypertension, dyslipidemia, diabetes mellitus, current smoking, congestive heart failure, prior history of stroke or transient ischemic attack (TIA), reperfusion therapies, and initial National Institutes of Health Stroke Scale (NIHSS) scores. We then performed a subgroup analysis to study the heterogeneity of effects associated with D-dimer levels and antithrombotic therapy on the risk of recurrent ischemic stroke according to age, sex, AF type, and the reperfusion therapy, which could influence D-dimer levels. *p* Values of <0.05 were considered statistically significant in the Cox proportional hazard regression analyses. Statistical analyses were performed using SPSS 23.0 (IBM Corporation, Armonk, NY, USA), SAS v. 9.4 (SAS Institute Inc., Cary, NC, USA), and R 3.3.1 (R Foundation for Statistical Computing, Vienna, Austria).

## 3. Results

### 3.1. Patient Characteristics

**Figure 1:**
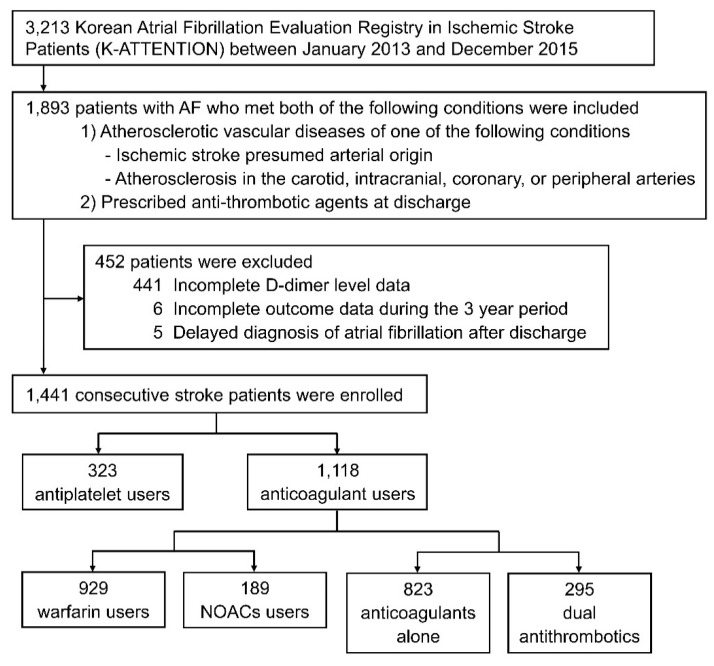
Flow chart of the study enrolment process.

Among the 3213 patients registered in the database, the study population was comprised of 1441 patients with AF-related stroke and atherosclerotic diseases (Figure 1). Patients with atherosclerosis demonstrated a history of susceptibility to risk factors such as hypertension, diabetes mellitus, and higher low-density lipoprotein (LDL) and lower high-density lipoprotein (HDL) cholesterol levels than the excluded patients without atherosclerosis (Table S1). There were no notable differences in age and sex between those who were included and excluded. The majority of AF-related stroke patients without atherosclerosis, who were excluded from the study, were treated with anticoagulants (*n* = 778/815, 95.5%).

The median follow-up duration was 17.6 months (interquartile range, 4.4–32.6 months). The mean age of the study population was 73.5 years (SD 9.7), and 750 (52.0%) patients were men. The clinical and biochemical characteristics of the patients who presented high (≥2 μg/mL) baseline D-dimer levels (*n* = 515, 35.7%) and low (<2 μg/mL) D-dimer levels (*n* = 926, 64.3%) are provided in Table 1 Compared to the low D-dimer group, the high D-dimer group was more likely to have higher CHA_2_DS_2_-VASc (Congestive heart failure, Hypertension, Age ≥75 years [doubled], Diabetes mellitus, Prior Stroke or TIA or Thromboembolism [doubled], Vascular disease, Age 65–74 years, Sex category) and initial NIHSS scores, and a higher incidence of sustained (persistent) AF instead of paroxysmal AF, along with valvular diseases, reperfusion therapies, and lower creatinine clearance. Intracranial atherosclerosis and a history of dyslipidemia were more common in the low D-dimer group than the high D-dimer group.

Table S2 contains information regarding the baseline characteristics of the patients who received antiplatelet (*n* = 323, 22.4%) and anticoagulant therapies (*n* = 1118, 77.6%) for the groups that underwent antithrombotic therapy. The anticoagulant group showed greater susceptibility for higher rates of sustained AF, a history of dyslipidemia, congestive heart failure, higher triglyceride levels, and lower initial NIHSS scores than the antiplatelet group. Carotid atherosclerosis or symptomatic atherosclerosis was more common in the antiplatelet group, and intracranial or peripheral atherosclerosis was higher in the anticoagulant group; however, the differences were not significant. The baseline D-dimer levels were similar in both treatment groups. Among those treated with NOACs (189 patients), dabigatran was the most used (in 74 patients). The NOACs used were identical for every group according to the baseline D-dimer levels (*p* = 0.148). Information regarding adherence to the prescribed medication after three months was available for patients at the largest center, and 84.1% (422/502) of these patients demonstrated high adherence to the same antithrombotic therapy.

### 3.2. Clinical Outcomes

**Figure 2:**
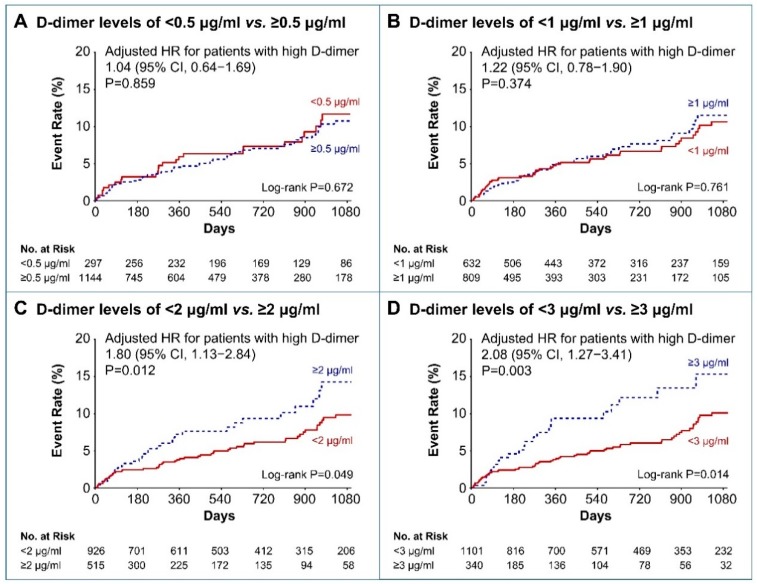
Kaplan–Meier curves and adjusted hazard ratios for recurrent ischemic stroke according to the baseline D-dimer levels. HR, hazard ratio; CI, confidence interval.

Overall, 87 patients were identified with recurrent ischemic stroke during the follow-up period (Table S3). There was no difference in the cumulative incidence of recurrent ischemic stroke between the patients after categorizing them into two groups based on the following D-dimer levels: 0.5 μg/mL and 1 μg/mL (log-rank *p* = 0.672 and *p* = 0.761, respectively; Figure 2A,B). However, patients with high baseline D-dimer levels (≥2 μg/mL) were at a higher risk for recurrent ischemic stroke than were those with low D-dimer levels (< 2 μg/mL; log-rank *p* = 0.049; Figure 2C). Similarly, those with high baseline D-dimer levels (≥3 μg/mL), were at a greater risk for recurrent ischemic stroke (log-rank *p* = 0.014; Figure 2D).

**Figure 3:**
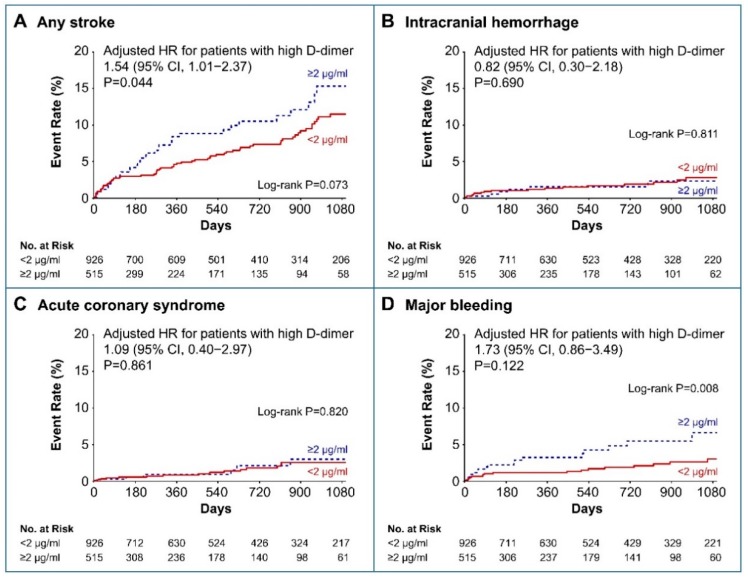
Kaplan–Meier curves and adjusted hazard ratios for secondary outcomes of (**A**) any stroke, (**B**) intracranial hemorrhage, (**C**) acute coronary syndrome, and (**D**) major bleeding according to the baseline D-dimer levels. HR, hazard ratio; CI, confidence interval.

Baseline high D-dimer levels ≥ 2 μg/mL and ≥ 3 μg/mL remained significantly associated with a higher risk for recurrent ischemic stroke after being adjusted for confounders using the multivariable Cox regression analyses (HR, 1.80; 95% CI, 1.13–2.84; *p* = 0.012; and HR, 2.08; 95% CI, 1.27–3.41; *p* = 0.003, respectively; Figure 2C,D). The risk for any stroke was also higher in patients with D-dimer levels ≥ 2 μg/mL than in those with D-dimer levels < 2 μg/mL after adjusting for confounders (HR, 1.54; 95% CI 1.01–2.37; *p* = 0.044; Figure 3A). The risk factors associated with ICH, ACS, and major bleeding were not significantly different between the high and low D-dimer groups (Figure 3B–D).

**Figure 4:**
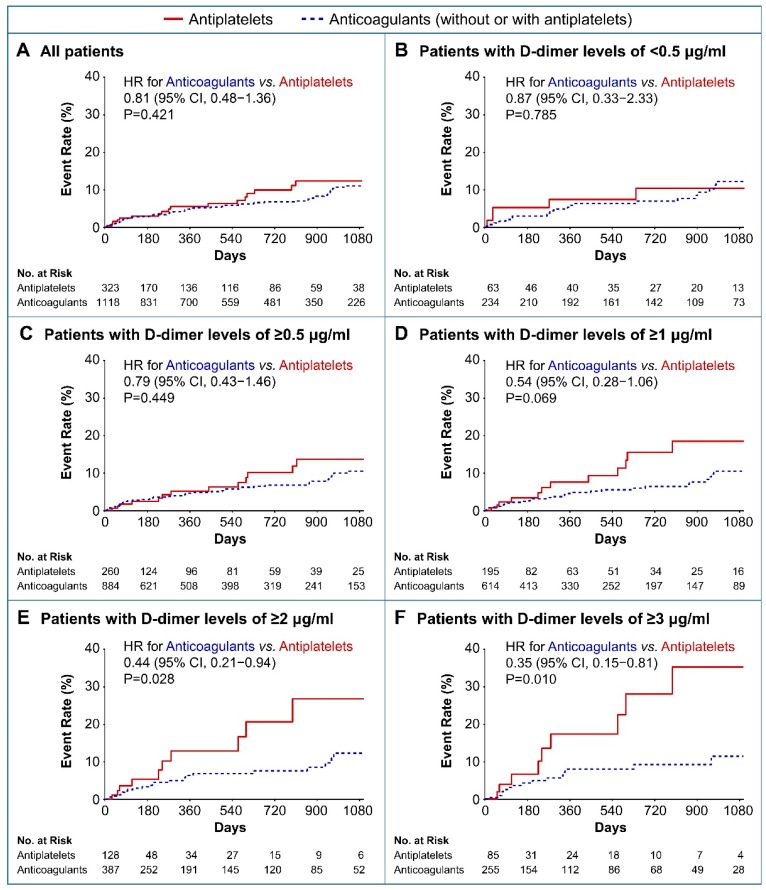
Kaplan–Meier curves for recurrent ischemic stroke according to antithrombotic therapy according to the baseline D-dimer levels. HR, hazard ratio; CI, confidence interval.

Figure 4 illustrates the results of the Kaplan–Meier analysis, which indicated that antiplatelets and OACs for secondary prevention of recurrent ischemic stroke, according to baseline D-dimer levels on the stroke index, were significantly different in patients having AF-related stroke and atherosclerotic diseases. Crude incidence rates of recurrent ischemic strokes were similar between the OAC group and the antiplatelet group among all subjects (3.95 per 100 person-years vs. 5.07 per 100 person-years; unadjusted HRs for the use of anticoagulants 0.81; 95% CI, 0.48–1.36; *p* = 0.421, respectively; Figure 4A and Table S3). However, compared to antiplatelets, OACs reduced the risk of recurrent ischemic stroke in patients with higher D-dimer levels (Figure 4B–F). High D-dimer level (≥2 μg/mL) patients, who were at a higher risk for recurrent ischemic stroke, demonstrated significantly lower risk of recurrent ischemic stroke in the OACs group than in the antiplatelet group (4.74 per 100 person-years vs. 11.10 per 100 person-years; unadjusted HRs for the use of OACs 0.44; 95% CI, 0.21–0.94; *p* = 0.028, respectively; Figure 4E and Table S3).

Regarding key secondary outcomes, there was no significant difference between the OAC and antiplatelet groups regarding the occurrence of any stroke, ICH, ACS, or major bleeding in all subjects (Figure S1 and Table S3). However, patients with high D-dimer levels (≥2 μg/mL) who were at a higher risk for recurrent ischemic stroke demonstrated a lower incidence of any stroke in the OAC group than in the antiplatelet group (5.37 per 100 person-years vs. 12.27 per 100 person-years; HR, 0.44; 95% CI, 0.220–0.93; *p* = 0.026, respectively; Figure S2 and Table S3). Incidences of the individual outcomes regarding ICH, ACS, and major bleeding were not significantly different between the OAC and antiplatelet groups (Figures S2 and S3, and Table S3).

**Figure 5:**
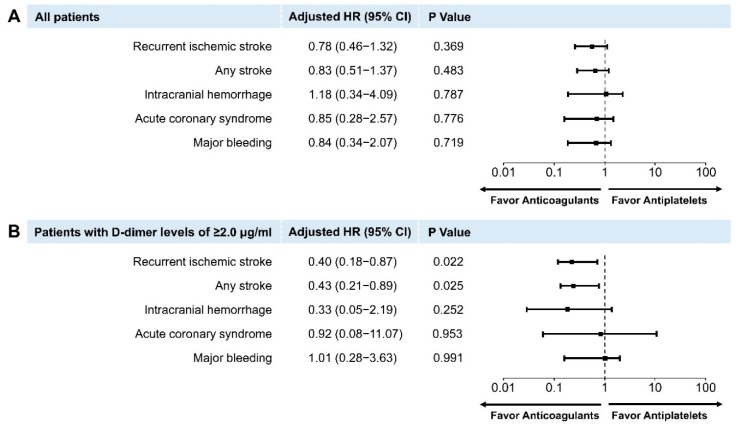
Cox proportional hazards regression analyses for primary and secondary outcomes. Adjusted hazard ratios for anticoagulant therapy compared with antiplatelet therapy in all patients (**A**) and patients with D-dimer levels of ≥ 2.0 μg/mL (**B**).

To better identify the effect of antithrombotic therapies on the risk for recurrent ischemic stroke in patients with AF-related stroke and atherosclerotic diseases, we adjusted for confounders using multivariable Cox regression analyses. In all patients with AF-related stroke and atherosclerotic diseases, there were no significant differences between the risks for recurrent ischemic stroke (HR, 0.78; 95% CI, 0.46–1.32; *p* = 0.369), any stroke (HR, 0.83; 95% CI, 0.51–1.37; *p* = 0.483), ICH (HR, 1.18; 95% CI, 0.34–4.09; *p* = 0.787), ACS (HR, 0.85; 95% CI, 0.28–2.57; *p* = 0.776), and major bleeding (HR, 0.84; 95% CI, 0.34–2.07; *p* = 0.719) among the antiplatelets (the reference group) and OAC groups (Figure 5A).

However, patients with high D-dimer levels (≥2 μg/mL) receiving anticoagulant therapy had a significantly lower risk for recurrent ischemic stroke (HR, 0.40; 95% CI, 0.18–0.87; *p* = 0.022; Figure 5B) than those receiving antiplatelet therapy. Similarly, those receiving anticoagulants were at a lower risk for any stroke among the secondary outcomes (HR, 0.43; 95% CI, 0.21–0.89; *p* = 0.025; Figure 5B). In contrast, there were no differences in the risks for ICH (HR, 0.33; 95% CI, 0.05–2.19; *p* = 0.252), ACS (HR, 0.92; 95% CI, 0.08–11.07; *p* = 0.953), and major bleeding (HR, 1.01; 95% CI, 0.28–3.63; *p* = 0.991) among those that underwent antiplatelet (the reference group) and OAC treatment (Figure 5B).

**Figure 6:**
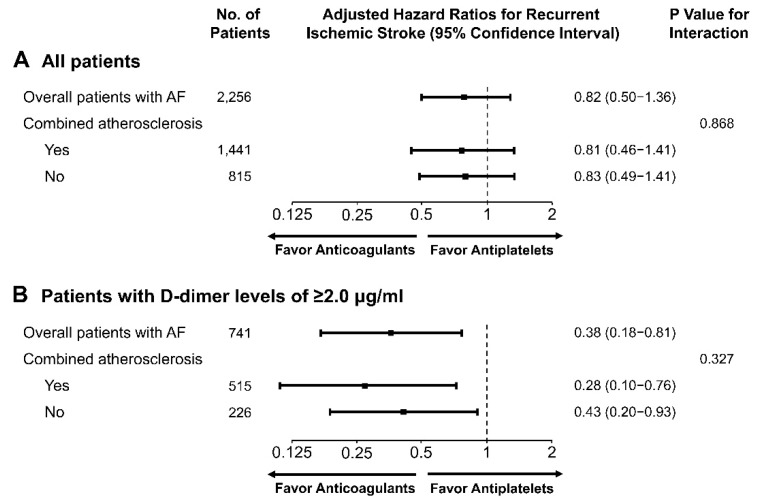
Adjusted hazard ratios for a comparison between anticoagulant and antiplatelet therapy in all patients with and without atherosclerosis (**A**) and a subgroup of patients with D-dimer levels ≥ 2.0 μg/mL (**B**). AF, atrial fibrillation.

We performed tests to analyze differences in the effects of anticoagulants to prevent recurrent ischemic stroke between patients with and without combined AF and atherosclerosis (included and excluded patients, respectively). Our results reported no significant difference in the effects of anticoagulants on the primary outcome in patients with and without combined AF and atherosclerosis (*p*_interaction_ = 0.868; Figure 6). The therapeutic effects of anticoagulant in patients with baseline D-dimer levels > 2 μg/mL, in comparison those of antiplatelet, were similar in patients with and without combined AF and atherosclerosis (*p*_interaction_ = 0.327; Figure 6).

**Figure 7:**
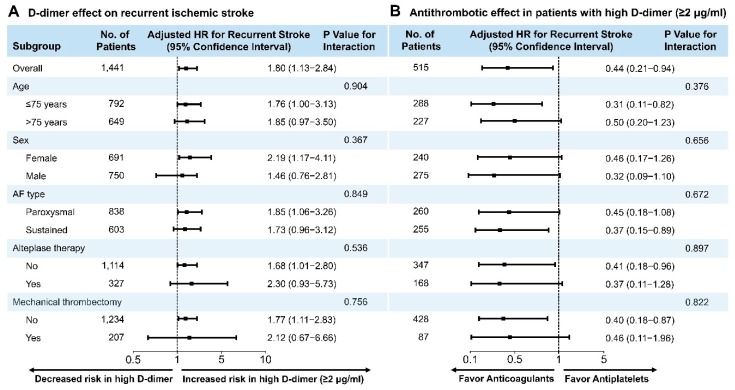
Subgroup analyses of recurrent stroke. The forest plot shows the differences in hazards ratio for high D-dimer levels (≥2.0 μg/mL) in all patients with combined AF and atherosclerosis (**A**) and a comparison between anticoagulant and antiplatelet therapy in patients with D-dimer levels ≥ 2.0 μg/mL (**B**). AF, atrial fibrillation.

Considering the presence of a significant difference among the AF types based on the D-dimer group, we evaluated a subgroup of patients with the paroxysmal and sustained AF separately. Among paroxysmal AF patients, the risk for recurrent stroke was higher in patients with higher D-dimer levels (≥2 μg/mL) than in those with relatively lower D-dimer levels (<2 μg/mL) after adjusting for confounders (HR, 2.39; 95% CI 1.26–4.52; *p* = 0.007; Table S4). Patients with sustained AF who presented high baseline D-dimer levels (≥2 μg/mL) and were administered anticoagulant therapy demonstrated a significantly lower risk for recurrent ischemic stroke than those who received antiplatelet therapy (HR, 0.17; 95% CI, 0.04–0.62; *p* = 0.007; Table S5). However, subgroup analyses revealed that the risk of high D-dimer levels (≥2.0 μg/mL) and beneficial effects of anticoagulant therapy in comparison with antiplatelet therapy on the risk of recurrent ischemic stroke in patients with high D-dimer levels (≥2.0 μg/mL) were uniform across all subgroups. This included both AF type and reperfusion therapy (Figure 7). The adjusted HRs for anticoagulant were not significant for male and female patients, older patients (age > 75 years), patients with paroxysmal AF, and patients who underwent reperfusion therapy; however, according to the subgroups there was no evidence of heterogeneity associated with the therapeutic effects of administering anticoagulants when compared with antiplatelet treatment (Figure 7).

We subsequently performed tests to analyze the differences between warfarin and NOACs as methods to prevent recurrent ischemic stroke and any stroke within the OAC groups. We also analyzed whether the dual antithrombotic therapy using both antiplatelets and OACs is more effective than OACs alone in patients having D-dimer levels of ≥2 μg/mL and at a higher risk for recurrent ischemic stroke. The mean time in the therapeutic range of warfarin was 50.9% (SD 24.0%). There were no differences in the risks of recurrent ischemic stroke (adjusted HR, 1.09; 95% CI, 0.35–3.30; *p* = 0.879) and any stroke (HR, 0.93; 95% CI, 0.31–2.78; *p* = 0.901) between the use of warfarin (the reference group) and NOACs in patients with D-dimer levels of ≥ 2 μg/mL (Figure S4). The risks of recurrent ischemic stroke (HR, 1.43; 95% CI, 0.57–3.57; *p* = 0.444) and any stroke (HR, 1.21; 95% CI, 0.49–2.94; *p* = 0.673) were also similar in patients treated with dual antithrombotic therapy with antiplatelets and OACs compared to those treated with OACs alone in patients with AF-related stroke and atherosclerotic diseases with high D-dimer levels (≥2 μg/mL) who were at a higher risk for recurrence (Figure S4).

## 4. Discussion

To the best of our knowledge, this is the first study to highlight the effects of D-dimer levels on the risk of recurrent stroke in a large, nationwide, population of patients with AF-related stroke and atherosclerotic diseases. Our study demonstrates that high D-dimer levels are independently associated with the risk of recurrent stroke in these patients. Furthermore, this is the first clinically applicable study on the efficacy and safety of different antithrombotic therapies according to baseline D-dimer levels. We found no difference between the effects of antiplatelets and anticoagulants in the prevention of recurrent ischemic stroke in all subjects or patients with low baseline D-dimer levels. However, we successfully demonstrated that compared with antiplatelets, anticoagulants were significantly associated with a lower risk of recurrent stroke in patients with D-dimer levels of ≥2 μg/mL. Therefore, high D-dimer levels would facilitate the identification of patients who are more likely to benefit from anticoagulants than antiplatelets for secondary prevention of stroke in those who have a high risk for recurrent stroke with both cardioembolic and non-cardioembolic causes.

D-dimer is typically formed post fibrin degradation and is, therefore, considered as a marker of hypercoagulable states associated with thrombus formation and resolution [5,13]. AF patients demonstrate increased D-dimer levels, which is related to the development of the left atrial thrombus [13,14]. Therefore, a causal association between high D-dimer levels and the risk for stroke is biologically plausible in AF patients [13,14]. Until recently, several studies had described the prognostic value of D-dimer levels for primary prevention of stroke in patients with AF [6,7]. Higher baseline D-dimer levels were related to a greater risk for stroke in AF patients.

Considering secondary prevention, a previous study reported that D-dimer levels were not typical indicators of recurrent stroke in patients with AF-related stroke [15]. However, this study was performed on relatively few patients (*n* = 382), and the evaluation of critical clinical outcomes using survival analysis over time was not systematically investigated [15]. Alternatively, the present study involved a comparatively larger cohort of patients with AF-related stroke, and we recently considered developed treatments for stroke such as mechanical thrombectomy and NOACs, and the various etiologies such as atherosclerotic disease that can accompany AF. This large-scale, clinically applicable, nationwide study was the first to demonstrate the prognostic value of high D-dimer levels for recurrent stroke in patients with AF-related stroke and atherosclerosis. Recently, a biomarker-based risk score for predicting stroke was proposed for AF patients [14,15]. Biomarkers, including high-sensitivity troponin, natriuretic peptide, and free fatty acid were independently associated with risk for stroke in AF patients [16,17,18]. Our results suggest D-dimer levels as a risk assessment biomarker to further refine recurrent stroke risk in patients with AF-related stroke and atherosclerosis [17].

Previous studies have reported that D-dimer levels were significantly higher in patients with cardioembolism than in those with other subtypes of ischemic stroke [19,20]. We noted that among patients with AF-related stroke, those with high D-dimer levels had a higher CHA_2_DS_2_-VASc score and were more likely to have sustained (persistent) AF and valvular heart diseases. High CHA2DS2-VASc score and valvular heart diseases are important predictors of recurrence in patients with stroke [21]. Furthermore, sustained AF could be associated with a higher risk of stroke or systemic embolism than paroxysmal AF [22,23]. Cardiogenic cerebral embolism in AF patients is typically caused by the formation of a thrombus in the left atrial appendage (LAA) [24]. Therefore, in stroke patients with AF, the function of LAA or the thrombus in LAA plays an important role in stroke recurrence [25]. As LAA function differs between paroxysmal and sustained AF, patients with sustained AF could have severer LAA blood stasis than those with paroxysmal AF [22,26]. Patients with a high baseline D-dimer level (≥2 μg/mL) demonstrate significantly higher incidences of sustained AF, which may be one of the primary etiological factors for the association of a high D-dimer level with frequent stroke recurrence in our study.

However, there were no significant differences in the thrombus and volume of the left atrium (LA) between the high and low D-dimer groups in our study, although high D-dimer group was more likely to have sustained AF. It is possible that the presence of an LA, particularly LAA, a thrombus, or dysfunction may have been underestimated since it was confirmed only by using transthoracic echocardiography (TTE) [27]. Previous studies have failed to detect LA thrombus using TTE until recently [28,29]. In other studies, the rate of LA thrombus detection using TTE is as low as approximately 1% in patients with AF-related stroke [30,31]. Since most patients with stroke in our study did not undergo transesophageal echocardiography (TEE) and LA thrombus was assessed only by TTE, it may appear that there is no difference among the D-dimer groups. However, a previous systematic review and meta-analysis reported that D-dimer had been significantly elevated in patients with a LA thrombus that was detected by TEE [32]. Therefore, if TEE was implemented, it would be possible that LA thrombus was observed more in the high D-dimer group, which had higher incidences of sustained AF.

Additionally, one of the reasons for the poor detection rates of LA thrombus could be due to the fact that it has been examined after thromboembolic events. TEE was better at detecting LA than TTE, particularly LAA thromus, and had a sensitivity and specificity of 99–100% [27,33]. However, LA thrombus was identified by TEE in only less than 50% of patients with newly recognized AF after the occurrence of an acute thromboembolic event, even if the TEE has 99–100% accuracy [34]. In our study, D-dimer levels were noted immediately in the emergency room at admission; however, TTE was typically evaluated after few days of hospitalization. TTE was examined after the embolism had already come off and followed by the development of the thromboembolic events, but also after several days of antithrombotic treatment to resolve the embolism. A study previously reported that short-term anticoagulation resolved over 80% of cases of thrombus on follow-up TEE [35]. Therefore, LA thrombus may have been detected as low as there is no difference between the D-dimer groups in this study.

Considering another possible mechanism by which high D-dimer increases the risk of recurrent stroke, our study revealed that patients with high D-dimer levels demonstrated poor creatinine clearance. Chronic kidney diseases may be predictive of strokes, which may be partially due to the limitations of pharmacotherapies, including limited effects of antithrombotic treatments [36]. Furthermore, a study previously demonstrated that D-dimer could be a useful marker of activated coagulation in patients with decreased renal function [37]. Therefore, the distinct relationship observed between D-dimer levels and stroke recurrence in our study could be attributed to the fact that patients with high D-dimer levels had lower creatinine clearance.

Since high D-dimer levels are an indicator for hypercoagulable states, anticoagulant administration would be relatively more effective than antiplatelet treatment [5,13]. However, no studies have directly identified or reported an antithrombotic therapeutic modality that is more effective in patients with AF-related stroke and atherosclerotic diseases with elevated D-dimer levels. Our results demonstrated that anticoagulants were more effective than antiplatelets in preventing recurrent stroke; additionally, there were no differences between the two groups concerning the risk for ICH and major extracranial bleeding in patients with D-dimer levels of ≥ 2 μg/mL. Therefore, this study highlights the importance of anticoagulant therapy in high D-dimer level patients with AF-related stroke and atherosclerotic diseases.

OACs are typically recommended for patients with AF-related stroke regardless of baseline D-dimer levels and the presence or absence of atherosclerotic diseases according to the current guidelines [3,38]. However, AF may not always be etiologically associated with ischemic stroke in patients with AF and atherosclerosis, and a study previously reported that one-sixth of strokes in patients with AF were unrelated to AF [39]. Moreover, the risk of bleeding due to OAC administration is significantly high, and it is also difficult to regulate the anticoagulation during blood sample measurement [40]. Therefore, physicians must carefully compare risks and benefits to determine the specific antithrombotic agent. Characteristically, several physicians are reluctant to prescribe OACs, due to which there are a large number of cases in actual clinical practice where antiplatelet agents have been administered to patients with AF-related stroke and atherosclerotic diseases [41,42]. Nevertheless, no studies have directly compared anticoagulation and antiplatelet therapy for secondary prevention of stroke in patients with AF and atherosclerotic diseases.

Previous studies have reported that 20–30% of patients admitted to hospitals with stroke and AF with an indication for OACs were taking antiplatelet agents alone until recently [41,42]. In our study, most AF-related stroke patients (95.5%) without atherosclerosis were treated with anticoagulants. However, antiplatelet therapy was administered in 22.4% of patients suffering from a combination of AF-related stroke and atherosclerotic diseases. In this real-world study, there were no significant differences between antiplatelets and anticoagulants to prevent recurrent stroke in patients with low baseline D-dimer levels. Although anticoagulation therapy in patients with AF-related stroke and atherosclerotic diseases may still be the most reasonable antithrombotic option, this study does not provide adequate evidence to suggest that anticoagulant therapy is more effective than antiplatelet therapy in patients with low D-dimer levels [3,38,40]. High baseline D-dimer levels might identify patients with AF and atherosclerotic diseases as those who are more likely to benefit from OACs than antiplatelets for secondary prevention of stroke in risk-benefit stratification.

Among OACs, it is reasonable to hypothesize that using an effective anticoagulant therapeutic strategy may suppress D-dimer levels to a greater degree, which could further reduce the risk of stroke in patients with elevated D-dimer levels [7,8,11]. Previous studies reported that NOACs were more efficient in reducing D-dimer levels than warfarin [7,8]. However, no studies have investigated the efficacy of NOACs versus warfarin in patients with AF-related stroke and atherosclerotic diseases. This study does not indicate that NOACs are more effective than warfarin. It only suggests that OAC therapy itself, which can reduce D-dimer levels, is more important than its numerous variations, which could be used to diminish D-dimer levels even further [43]. However, considering the small proportion of patients in this study who underwent NOAC administration, it is imperative to conduct additional large-scale randomized controlled trials (RCTs) in the future.

Our study has several limitations; therefore, the findings must be interpreted with caution. First, as this is a prospectively registered observational study that involved retrospective data analysis and since there was no subject randomization, our data is prone to possible residual bias and significant methodologic shortcomings. Second, we did not collect detailed information on the changes in D-dimer levels after antithrombotic treatment during follow-up. Third, D-dimer value could change over time and be affected by anticoagulation or thrombolytic therapy [7,8,11]. There are different methods and time points to measure D-dimer levels, which may lead to substantial variations in the results. Therefore, the clinical prognostic value of D-dimer levels could not be confirmed. Fourth, we did not consider the potential effects of deep vein thrombosis, pulmonary thromboembolism, malignancy, and hematological diseases that could affect D-dimer levels [13]. In case the D-dimer level is elevated in patients with these diseases, and considering the preference of OAC therapeutic strategy, this may lead to the formation of a residual bias associated with these diseases [44]. Fifth, the possibility that LA, especially LAA, thrombus or dysfunction could be the primary cause of cardiogenic cerebral embolism in patients with AF may have been severely underestimated, since it was confirmed only by using TTE, but not TEE [33]. Sixth, we did not account for the following important risk factors that can lead to atherosclerosis and AF: emotional stress, diet, and physical inactivity [12,45,46]. Finally, we did not comply with the prescribed antithrombotics and ascertained the duration of using these antithrombotics via direct methods. Furthermore, information regarding adherence to medication after three months was available for the study population in the largest center only. However, approximately 84% of patients with available medication adherence information reported high adherence with the same antithrombotic therapy.

## 5. Conclusions

Our findings suggested that baseline D-dimer levels were associated with risk for recurrent stroke in patients with AF-related stroke and atherosclerotic diseases. Therefore, these results suggest that high D-dimer levels were a risk assessment biomarker for recurrent stroke in patients with AF-related stroke and atherosclerotic diseases. Among patients with high D-dimer levels (≥2 μg/mL) at higher risk for recurrent ischemic stroke, OAC therapy was relatively more effective in reducing the risk for recurrent stroke than antiplatelet therapy. These results support the use of OACs in patients with AF-related stroke and atherosclerotic diseases at high risk for recurrent stroke based on the risk assessment of baseline D-dimer levels. In addition, we found no difference between NOACs and warfarin as well as the dual antithrombotics and OACs alone in secondary prevention of recurrent ischemic stroke in patients with AF-related stroke and atherosclerotic diseases, suggesting that these may be reasonable therapeutic strategies. These findings require further investigation and confirmation in prospective RCTs.

## Figures and Tables

**Table 1:** Baseline clinical and biochemical characteristics according to the baseline D-dimer levels.

	Patients with D-Dimer Levels of < 2.0 μg/mL (*n* = 926)	Patients with D-Dimer Levels of ≥ 2.0 μg/mL (*n* = 515)	Total (*n* = 1441)	*p* Value
Age, years (mean ± SD)	73.6 ± 9.6	73.2 ± 9.8	73.5 ± 9.7	0.454
Male, *n* (%)	475 (51.3)	275 (53.4)	750 (52.0)	0.477
Valvular AF, *n* (%)	23 (2.5)	8 (1.6)	31 (2.2)	0.328
AF type, *n* (%)				<0.001
Paroxysmal AF	578 (62.4)	260 (50.5)	838 (58.2)	
Sustained AF	348 (37.6)	255 (49.5)	603 (41.8)	
Body mass index	23.2 ± 3.3	23.4 ± 3.4	23.3 ± 3.3	0.465
	**History of Risk Factors, *n* (%)**			
Hypertension	650 (70.2)	376 (73.0)	1026 (71.2)	0.284
Diabetes mellitus	268 (28.9)	142 (27.6)	410 (28.5)	0.623
Dyslipidemia	217 (23.4)	96 (18.6)	313 (21.7)	0.041
Congestive heart failure	34 (3.7)	22 (4.3)	56 (3.9)	0.673
Current smoking	142 (15.3)	62 (12.0)	204 (14.2)	0.101
Prior stroke or TIA	276 (29.8)	178 (34.6)	454 (31.5)	0.071
	**Biochemical Variables (mean ± SD)**			
D-dimer, μg/mL	0.8 ± 0.5	6.1 ± 6.5	2.7 ± 4.6	<0.001
LDL-C, mg/dL	98.4 ± 33.9	97.6 ± 34.6	98.1 ± 34.1	0.708
Triglyceride, mg/dL	99.4 ± 63.1	92.4 ± 48.2	96.9 ± 58.3	0.019
HDL-C, mg/dL	48.3 ± 19.2	47.0 ± 17.1	47.8 ± 18.5	0.186
Glycated hemoglobin, %	6.1 ± 2.0	5.9 ± 1.0	6.0 ± 1.7	0.078
Admission glucose, mg/dL	139.2 ± 81.2	137.8 ± 48.5	138.7 ± 71.2	0.687
Creatinine clearance, mL/min	65.6 ± 28.0	56.3 ± 27.8	62.3 ± 28.3	<0.001
Pre-stroke mRS, median (IQR)	0 (0;1)	0 (0;2)	0 (0; 1)	0.054
Initial NIHSS, median (IQR)	6 (2;13)	13 (6;18)	9 (2; 15)	<0.001
Intravenous alteplase, *n* (%)	159 (17.2)	168 (32.6)	327 (22.7)	<0.001
Mechanical thrombectomy, *n* (%)	120 (13.0)	87 (16.9)	207 (14.4)	0.050
CHA_2_DS_2_-VASc score, median (IQR)	5 (4; 6)	5 (4;6)	5 (4;6)	0.001
CHA_2_DS_2_-VASc ≥ 5, *n* (%)	552 (59.6)	372 (72.2)	924 (64.1)	<0.001
AIS presumed arterial origin, *n* (%)	276 (29.8)	162 (31.5)	438 (30.4)	0.553
	**Large Artery Atherosclerosis, *n* (%)**			
Carotid atherosclerosis	260 (31.7)	154 (32.7)	414 (32.1)	0.772
Intracranial atherosclerosis	648 (78.0)	314 (65.4)	962 (73.4)	<0.001
Coronary atherosclerosis	144 (15.6)	69 (13.4)	213 (14.8)	0.305
Peripheral atherosclerosis	13 (1.4)	8 (1.6)	21 (1.5)	1.000
	Transthoracic Echocardiographic Findings *			
LA thrombus, *n* (%)	11 (1.3)	11 (2.3)	22 (1.7)	0.259
LA volume index, mL/m^2^	66.0 ± 24.8	67.0 ± 24.9	66.3 ± 24.8	0.493
LVEF < 50%, *n* (%)	122 (14.5)	74 (15.6)	196 (14.9)	0.677
LV diastolic dysfunction, *n* (%)	596 (71.2)	343 (72.3)	939 (71.6)	0.727
Moderate–severe AR, *n* (%)	9 (1.1)	13 (2.7)	22 (1.7)	0.042
Moderate–severe MR, *n* (%)	26 (3.1)	23 (4.9)	49 (3.8)	0.149

* Data for 131 patients (90 patients in the low D-dimer group and 41 patients in the high D-dimer group) was unavailable. AF, atrial fibrillation; TIA, transient ischemic attack; SD, standard deviation; LDL-C, low-density lipoprotein cholesterol; HDL-C, high-density lipoprotein cholesterol; mRS, modified Rankin Scale; NIHSS, National Institutes of Health Stroke Scale; IQR, interquartile ranges; AIS, acute ischemic stroke; LA, left atrium; LVEF, left ventricular ejection fraction; LV, left ventricle; AR, aortic regurgitation; MR, mitral regurgitation.

## Supplementary Materials

The following are available online at https://www.mdpi.com/2077-0383/8/9/1457/s1, Figure S1: Kaplan–Meier curves and adjusted hazard ratios for secondary outcomes, Figure S2: Kaplan–Meier curves for different types of stroke in all patients, Figure S3: Kaplan–Meier curves for different types of stroke in patients with D-dimer levels of ≥ 2.0 μg/mL, Figure S4: The cumulative incidence and adjusted hazard ratios according to antithrombotic therapy, Table S1: Baseline clinical and biochemical characteristics in patients included and excluded, Table S2: Baseline characteristics according to antithrombotic therapy, Table S3: Event rates and association estimates from Cox proportional hazard modeling according to antithrombotic therapy, Table S4: Effects of high D-dimer levels on outcomes in patients with paroxysmal and sustained atrial fibrillation, Table S5: Hazard ratios for anticoagulant therapy in patients with the paroxysmal and sustained atrial fibrillation who presented high (≥2 μg/mL) baseline D-dimer levels.
