# Supplementary material for: Baseline D-Dimer Levels as a Risk Assessment Biomarker for Recurrent Stroke in Patients with Combined Atrial Fibrillation and Atherosclerosis
Source: J Clin Med. 2019 Sep 13;8(9):1457. doi: 10.3390/jcm8091457 (PMC6780256; doi:10.3390/jcm8091457)
Supplement: Supplementary file 1 [file jcm-08-01457-s001.pdf]

# SUPPLEMENTAL MATERIAL

## Supplementary Tables

**Table S1.** Baseline clinical and biochemical characteristics of both included and excluded patients.

| | Included patients (n=1,441) | Excluded patients (n=1,772) | Total (n=3,213) | *p* value |
|---|---|---|---|---|
| Age, years (mean ± SD) | 73.5 ± 9.7 | 73.7 ± 10.0 | 73.6 ± 9.9 | 0.541 |
| Male, n (%) | 750 (52.0) | 900 (50.8) | 1650 (51.4) | 0.501 |
| Body mass index | 23.3 ± 3.3 | 23.3 ± 3.5 | 23.3 ± 3.4 | 0.677 |
| **History of risk factors, n (%)** | | | | |
| Hypertension | 1026 (71.2) | 1201 (67.8) | 2227 (69.3) | 0.040 |
| Diabetes mellitus | 410 (28.5) | 446 (25.2) | 856 (26.6) | 0.040 |
| Dyslipidemia | 313 (21.7) | 455 (25.7) | 768 (23.9) | 0.010 |
| Congestive heart failure | 56 (3.9) | 85 (4.8) | 141 (4.4) | 0.243 |
| Current smoking | 204 (14.2) | 226 (12.8) | 430 (13.4) | 0.267 |
| Prior stroke or TIA | 454 (31.5) | 604 (34.1) | 1058 (32.9) | 0.131 |
| **Biochemical variables (mean ± SD)** | | | | |
| LDL-C, mg/dL | 98.1 ± 34.1 | 93.7 ± 36.1 | 95.8 ± 35.2 | 0.001 |
| Triglyceride, mg/dL | 96.9 ± 58.3 | 98.4 ± 62.9 | 97.7 ± 60.7 | 0.511 |
| HDL-C, mg/dL | 47.8 ± 18.5 | 53.2 ± 26.7 | 50.6 ± 23.3 | <0.001 |
| Glycated hemoglobin, % | 6.0 ± 1.7 | 6.1 ± 2.0 | 6.1 ± 1.9 | 0.190 |
| Admission glucose, mg/dL | 138.7 ± 71.2 | 142.2 ± 69.5 | 140.5 ± 70.3 | 0.176 |
| Creatinine clearance, mL/min | 62.3 ± 28.3 | 61.6 ± 29.9 | 61.9 ± 29.2 | 0.527 |
| Pre-stroke mRS, median (IQR) | 0 (0;1) | 0 (0;1) | 0 (0;1) | 0.098 |
| Initial NIHSS, median (IQR) | 9 (2;15) | 8 (2;15) | 8 (2;15) | 0.625 |
| Intravenous alteplase, n (%) | 327 (22.7) | 393 (22.2) | 720 (22.4) | 0.760 |
| Mechanical thrombectomy, n (%) | 207 (14.4) | 165 (9.3) | 372 (11.6) | <0.001 |
| $CHA_2DS_2$-VASc score, median (IQR) | 5 (4; 6) | 5 (4; 6) | 5 (4; 6) | 0.238 |

SD, standard deviation; TIA, transient ischemic attack; LDL-C, low-density lipoprotein cholesterol; HDL-C, high-density lipoprotein cholesterol; mRS, modified Rankin Scale; IQR, interquartile ranges; NIHSS, National Institutes of Health Stroke Scale.

**Table S2.** Baseline characteristics according to antithrombotic therapy.

| | Antiplatelets (n=323) | Anticoagulants (n=1,118) | Total (n=1,441) | *p* value |
|---|---|---|---|---|
| Age, years (mean ± SD) | 73.8 ± 9.0 | 73.4 ± 9.8 | 73.5 ± 9.7 | 0.509 |
| Male, n (%) | 167 (51.7) | 583 (52.1) | 750 (52.0) | 0.938 |
| AF type, n (%) | | | | <0.001 |
| Paroxysmal AF | 248 (76.8) | 590 (52.8) | 838 (58.2) | |
| Sustained AF | 75 (23.2) | 528 (47.2) | 603 (41.8) | |
| Body mass index | 23.3 ± 3.3 | 23.3 ± 3.3 | 23.3 ± 3.3 | 0.951 |
| **History of risk factors, n (%)** | | | | |
| Hypertension | 225 (69.7) | 801 (71.6) | 1026 (71.2) | 0.532 |
| Diabetes mellitus | 93 (28.8) | 317 (28.4) | 410 (28.5) | 0.933 |
| Dyslipidemia | 46 (14.2) | 267 (23.9) | 313 (21.7) | <0.001 |
| Congestive heart failure | 5 (1.5) | 51 (4.6) | 56 (3.9) | 0.021 |
| Current smoking | 46 (14.2) | 158 (14.1) | 204 (14.2) | 1.000 |
| Prior stroke or TIA | 104 (32.2) | 350 (31.3) | 454 (31.5) | 0.813 |
| **Biochemical variables (mean ± SD)** | | | | |
| D-dimer, µg/mL | 3.0 ± 5.0 | 2.6 ± 4.5 | 2.7 ± 4.6 | 0.235 |
| LDL-C, mg/dL | 96.9 ± 34.5 | 98.5 ± 34.0 | 98.1 ± 34.1 | 0.473 |
| Triglyceride, mg/dL | 92.1 ± 48.8 | 98.3 ± 60.7 | 96.9 ± 58.3 | 0.056 |
| HDL-C, mg/dL | 47.5 ± 17.1 | 47.9 ± 18.9 | 47.8 ± 18.5 | 0.699 |
| Glycated hemoglobin, % | 5.9 ± 1.2 | 6.0 ± 1.9 | 6.0 ± 1.7 | 0.219 |
| Admission glucose, mg/dL | 139.0 ± 50.3 | 138.6 ± 76.2 | 138.7 ± 71.2 | 0.924 |
| Creatinine clearance, mL/min | 62.2 ± 27.5 | 62.3 ± 28.5 | 62.3 ± 28.3 | 0.960 |
| Pre-stroke mRS, median (IQR) | 0 (0;3) | 0 (0;1) | 0 (0;1) | <0.001 |
| Initial NIHSS, median (IQR) | 10 (3;16) | 9 (2;15) | 9 (2;15) | 0.031 |

| | | | | |
|---|---|---|---|---|
| Intravenous alteplase, n (%) | 77 (23.8) | 250 (22.4) | 327 (22.7) | 0.629 |
| Mechanical thrombectomy, n (%) | 45 (13.9) | 162 (14.5) | 207 (14.4) | 0.871 |
| $CHA_2DS_2$-VASc score, median (IQR) | 5 (4; 6) | 5 (4; 6) | 5 (4; 6) | 0.186 |
| AIS presumed arterial origin, n (%) | 101 (31.3) | 337 (30.1) | 438 (30.4) | 0.750 |
| Symptomatic atherosclerosis, n (%) | 239 (74.0) | 774 (69.2) | 1013 (70.3) | 0.114 |
| **Large artery atherosclerosis, n (%)** | | | | |
| Carotid atherosclerosis | 109 (36.2) | 305 (30.8) | 414 (32.1) | 0.093 |
| Intracranial atherosclerosis | 213 (69.4) | 749 (74.6) | 962 (73.4) | 0.082 |
| Coronary atherosclerosis | 42 (13.0) | 171 (15.3) | 213 (14.8) | 0.351 |
| Peripheral atherosclerosis | 1 (0.3) | 20 (1.8) | 21 (1.5) | 0.091 |
| OACs with antiplatelets, n (%) | - | 295 (26.4) | 295 (26.4) | - |

AF, atrial fibrillation; TIA, transient ischemic attack; SD, standard deviation; LDL-C, low-density lipoprotein cholesterol; HDL-C, high-density lipoprotein cholesterol; mRS, modified Rankin Scale; NIHSS, National Institutes of Health Stroke Scale; IQR, interquartile ranges; AIS, acute ischemic stroke; LA, left atrium; LVEF, left ventricular ejection fraction; LV, left ventricle.

**Table S3.** Event rates and association estimates from Cox proportional hazard modeling according to antithrombotic therapy in all patients and patients with D-dimer levels of ≥2.0 μg/mL.

| | All patients | | | Patients with D-dimer levels of ≥2.0 μg/mL | | |
|---|---|---|---|---|---|---|
| **Clinical outcomes** | **Antiplatelets (n=323)** | **OACs (n=1,118)** | **HR (95% CI); *p* value*** | **Antiplatelets (n=128)** | **OACs (n=387)** | **HR (95% CI); *p* value*** |
| **Primary outcome** | | | | | | |
| Recurrent ischemic stroke | | | 0.81 (0.48-1.36); 0.421 | | | 0.44 (0.21-0.94); 0.028 |
| Number of events | 18 | 69 | | 10 | 23 | |
| Incidence per 100 person-years | 5.07 | 3.95 | | 11.10 | 4.74 | |
| **Secondary outcomes** | | | | | | |
| Any stroke | | | 0.86 (0.53-1.40); 0.541 | | | 0.44 (0.22-0.93); 0.026 |
| Number of events | 20 | 81 | | 11 | 26 | |
| Incidence per 100 person-years | 5.64 | 4.65 | | 12.27 | 5.37 | |
| Intracranial hemorrhage | | | 0.99 (0.33-2.92); 0.979 | | | 0.31 (0.05-1.88); 0.203 |
| Number of events | 4 | 18 | | 2 | 3 | |
| Incidence per 100 person-years | 1.08 | 1.00 | | 2.15 | 0.59 | |
| Acute coronary syndrome | | | 0.84 (0.28-2.52); 0.756 | | | 0.94 (0.11-8.13); 0.953 |
| Number of events | 4 | 16 | | 1 | 5 | |
| Incidence per 100 person-years | 1.09 | 0.89 | | 1.07 | 1.00 | |
| Major bleeding | | | 1.05 (0.44-2.54); 0.910 | | | 1.03 (0.30-3.62); 0.959 |
| Number of events | 6 | 28 | | 3 | 14 | |
| Incidence per 100 person-years | 1.62 | 1.56 | | 3.20 | 2.79 | |

* Unadjusted hazard ratios and *p* values for anticoagulant therapy compared to antiplatelet therapy.
OACs, oral anticoagulants; HR, hazard ratio; CI, confidence interval.

**Table S4.** Effects of high D-dimer levels (≥2.0 μg/mL) on primary and secondary outcomes in patients with paroxysmal and sustained atrial fibrillation (AF).

| Outcomes | Patients with paroxysmal AF | | Patients with sustained AF | |
|---|---|---|---|---|
| | Adjusted HR (95% CI) | *p* value | Adjusted HR (95% CI) | *p* value |
| **Primary outcome** | | | | |
| Recurrent ischemic stroke | 2.39 (1.26−4.52) | 0.007 | 1.35 (0.69−2.63) | 0.373 |
| **Secondary outcomes** | | | | |
| Any stroke | 2.26 (1.22−4.20) | 0.009 | 1.17 (0.64−2.13) | 0.610 |
| Intracranial hemorrhage | 1.62 (0.25−10.37) | 0.607 | 0.43 (0.11−1.64) | 0.223 |
| Acute coronary syndrome | 0.92 (0.24−3.45) | 0.903 | 2.10 (0.30−14.74) | 0.453 |
| Major bleeding | 2.14 (0.77−5.92) | 0.143 | 1.73 (0.64−4.66) | 0.276 |

HR, hazard ratio; CI, confidence interval.

**Table S5.** Adjusted hazard ratios for anticoagulant therapy compared with antiplatelet therapy in patients with the paroxysmal and sustained atrial fibrillation (AF) who presented high (≥2 μg/mL) baseline D-dimer levels.

| Outcomes | Patients with paroxysmal AF | | Patients with sustained AF | |
|---|---|---|---|---|
| | Adjusted HR (95% CI) | *p* value | Adjusted HR (95% CI) | *p* value |
| **Primary outcome** | | | | |
| Recurrent ischemic stroke | 0.52 (0.18−1.52) | 0.237 | 0.17 (0.04−0.62) | 0.007 |
| **Secondary outcomes** | | | | |
| Any stroke | 0.45 (0.16−1.25) | 0.128 | 0.24 (0.07−0.86) | 0.028 |
| Intracranial hemorrhage | NA | 0.467 | NA | 0.967 |
| Acute coronary syndrome | NA | 0.982 | 0.14 (0.01−8.01) | 0.187 |
| Major bleeding | 0.64 (0.13−3.18) | 0.590 | NA | 0.986 |

HR, hazard ratio; CI, confidence interval; NA, not available.

## Supplementary Figures

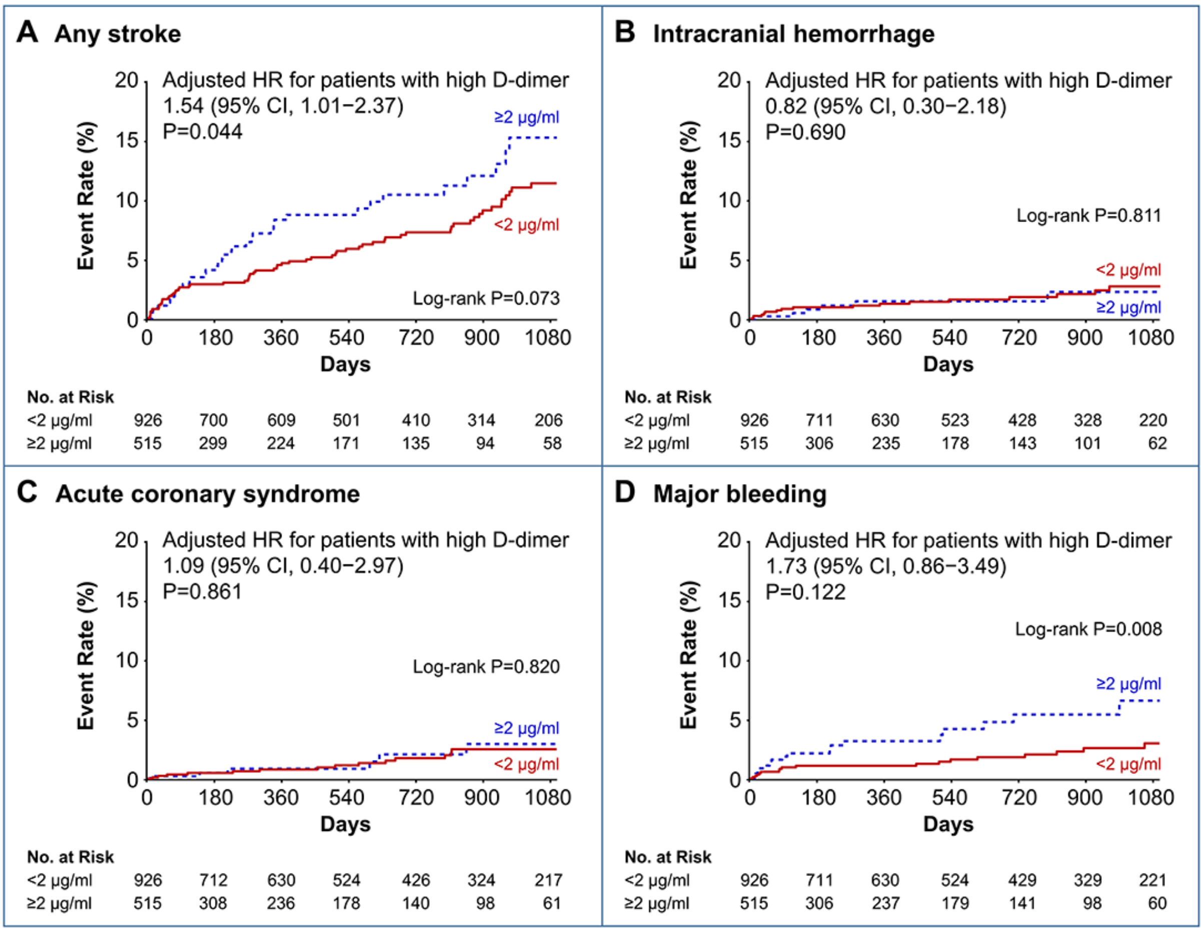


**Figure S1.** Kaplan-Meier curves and adjusted hazard ratios for secondary outcomes of (A) any stroke, (B) intracranial hemorrhage, (C) acute coronary syndrome, and (D) major bleeding according to the baseline D-dimer levels. HR, hazard ratio; CI, confidence interval.

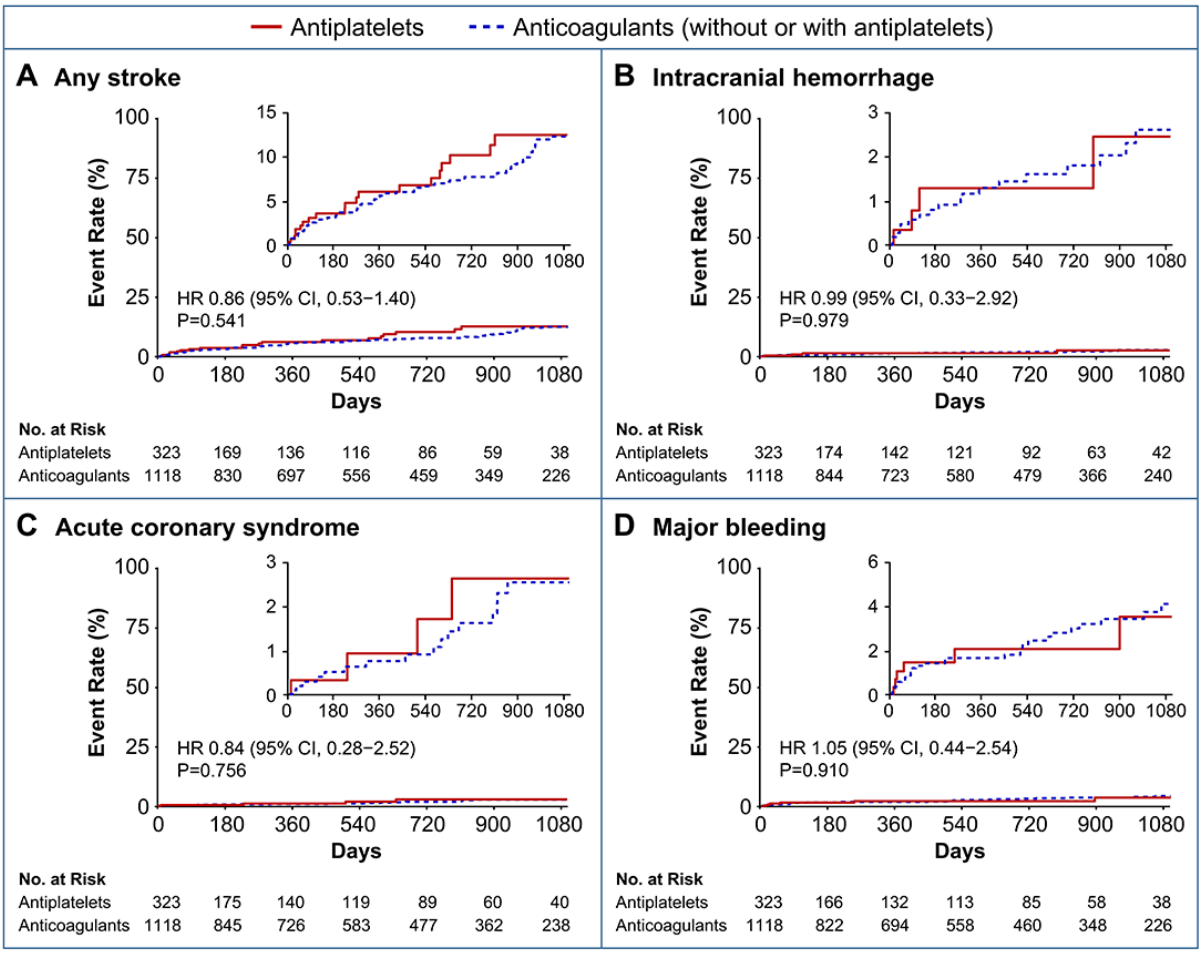


**Figure S2.** Kaplan-Meier curves for different types of stroke in all patients. Kaplan-Meier curves for (A) any stroke, (B) intracranial hemorrhage, (C) acute coronary syndrome, and (D) major bleeding according to antithrombotic therapy in all patients. HR, hazard ratio; CI, confidence interval.

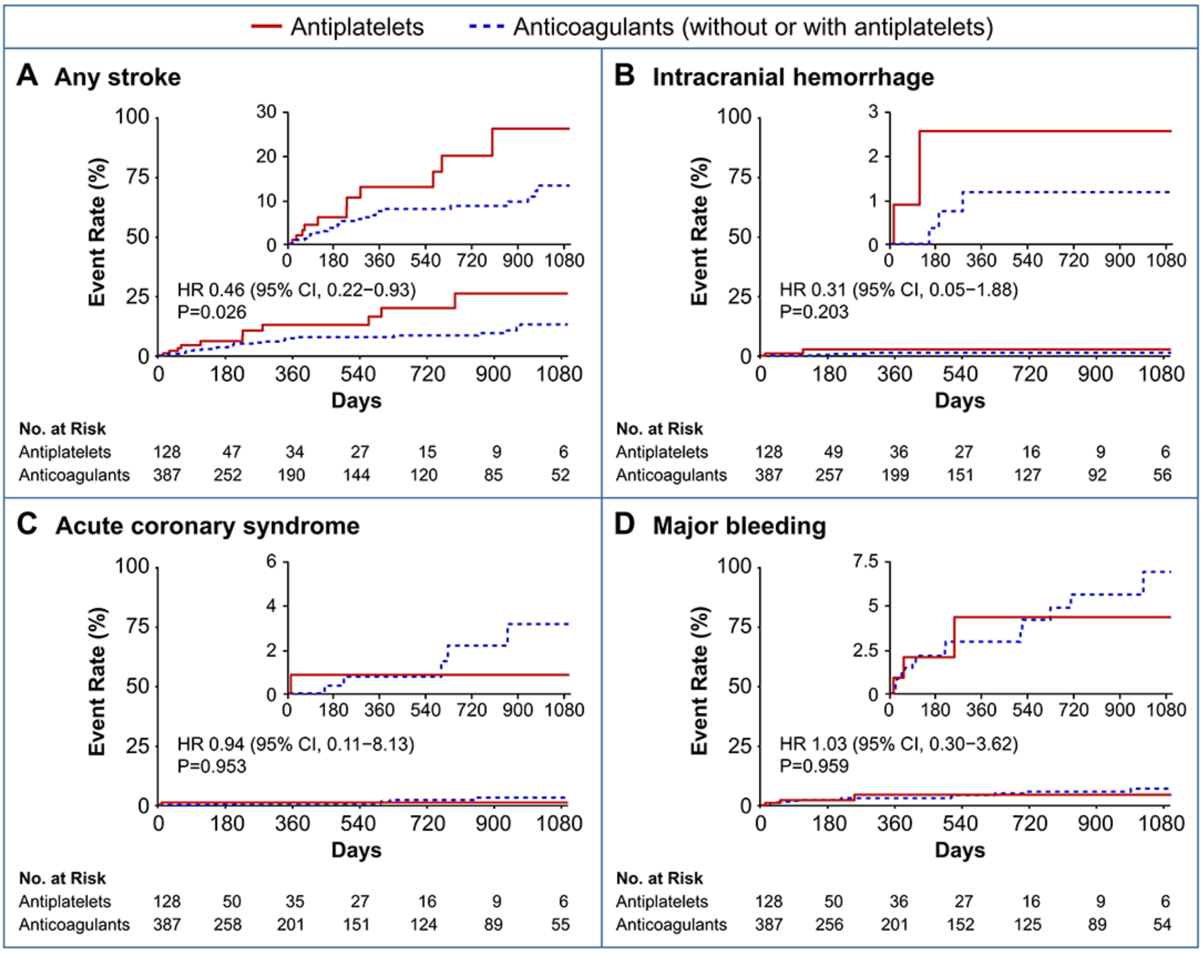


**Figure S3.** Kaplan-Meier curves for different types of stroke in patients with D-dimer levels of ≥2.0 μg/mL. Kaplan-Meier curves for (A) any stroke, (B) intracranial hemorrhage, (C) acute coronary syndrome, and (D) major bleeding according to antithrombotic therapy in patients with D-dimer levels of ≥2.0 μg/mL. HR, hazard ratio; CI, confidence interval.

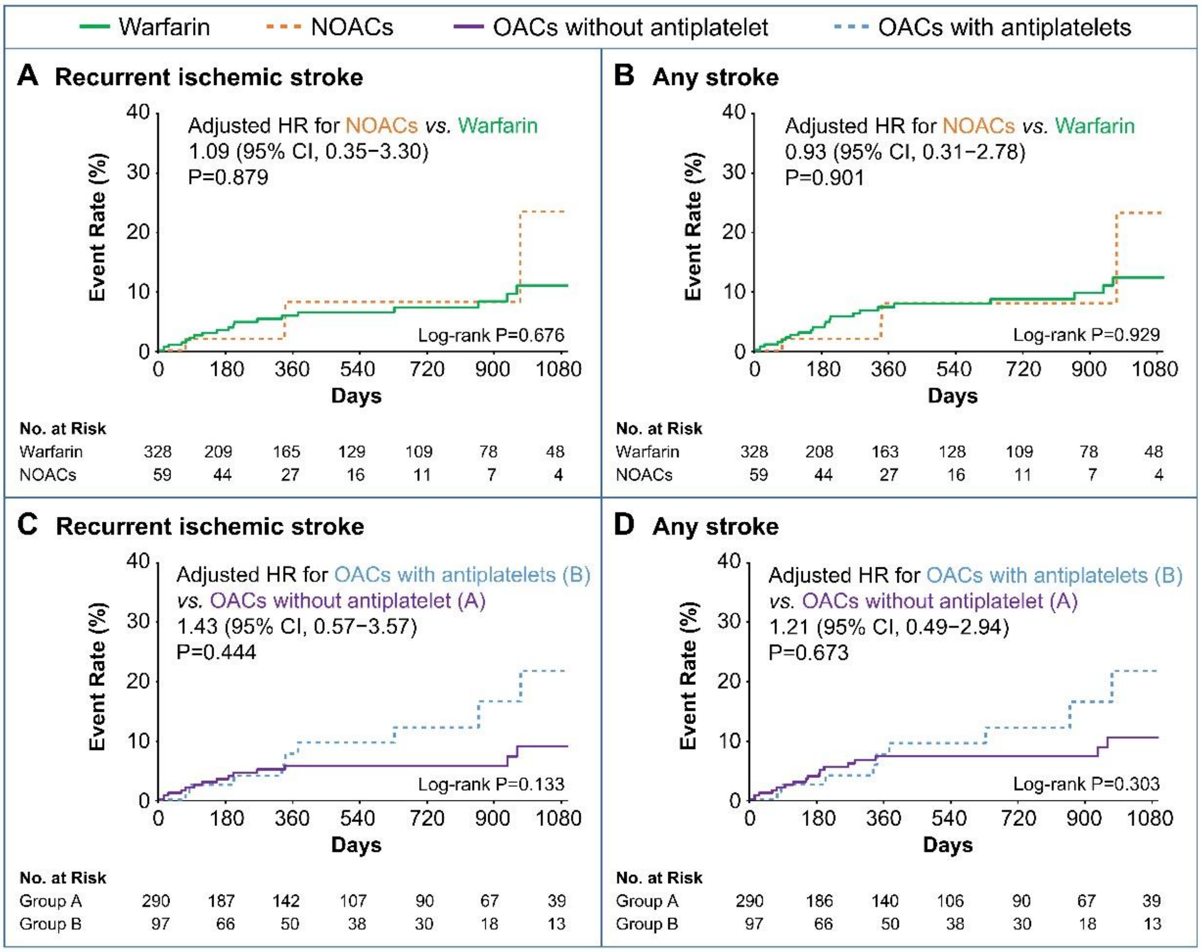


**Figure S4.** The cumulative incidence and adjusted hazard ratios of recurrent ischemic stroke (A and C) and any stroke (B and D) according to antithrombotic therapy in patients with D-dimer levels of ≥2.0 μg/mL. NOAC, non-vitamin K antagonist oral anticoagulants; OAC, oral anticoagulants; HR, hazard ratio; CI, confidence interval.
